# Supplementary material for: SMARCB1 loss activates patient-specific distal oncogenic enhancers in malignant rhabdoid tumors
Source: Nat Commun. 2023 Dec 1;14:7762. doi: 10.1038/s41467-023-43498-3 (PMC10692191; doi:10.1038/s41467-023-43498-3)
Supplement: Supplementary file 1 — Supplementary Information [file 41467_2023_43498_MOESM1_ESM.pdf]

Supplementary Fig. 1

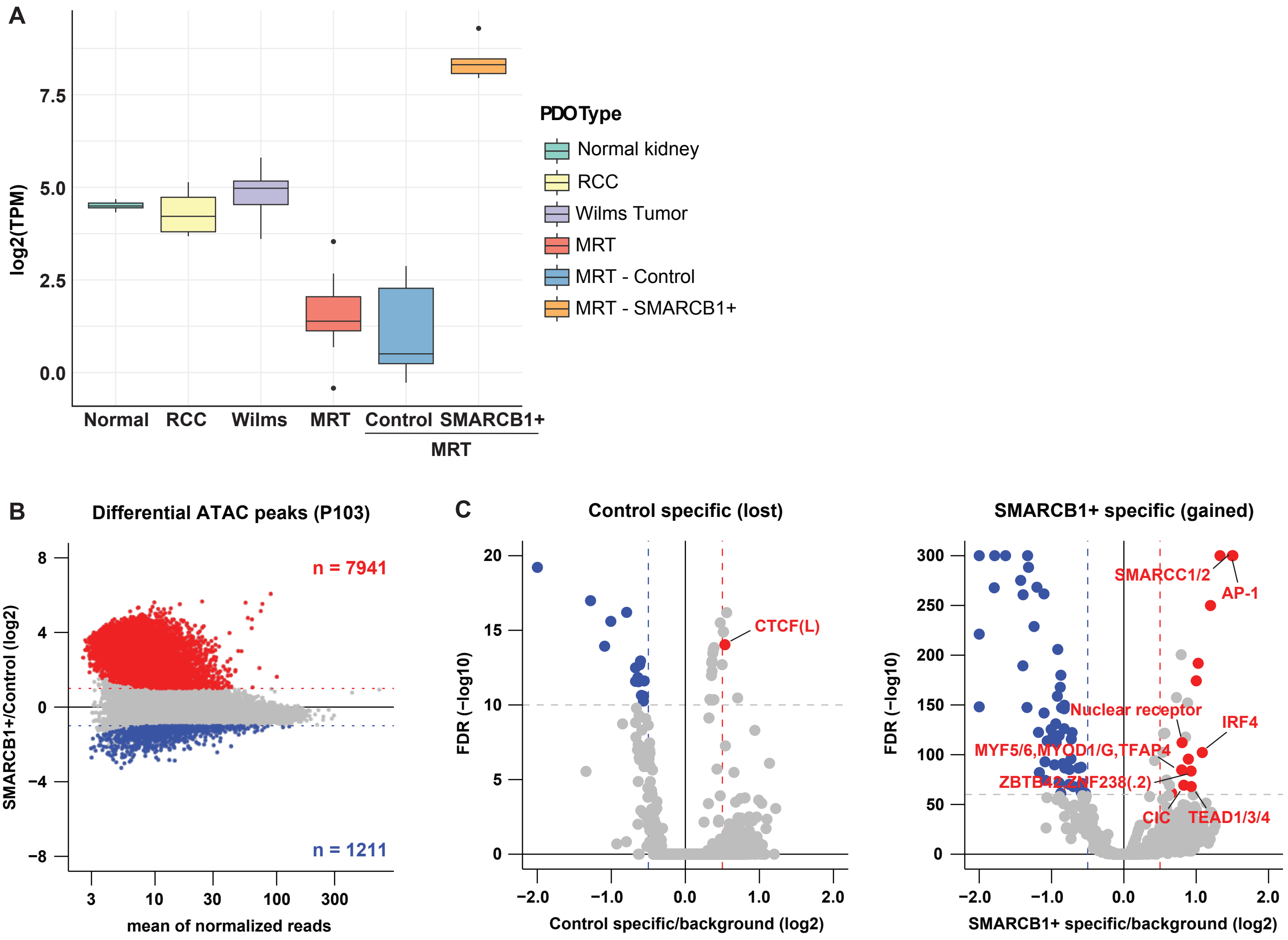

D Top 5 most significant biological processes (P103)

ID	Biological process	Fold change	FDR
**Control specific (lost) term:**			
GO:0050777	negative regulation of immune response	2.61	1.08E-04
GO:0045814	negative regulation of gene expression, epigenetic	2.71	1.89E-04
GO:0006334	nucleosome assembly	2.43	8.16E-04
GO:0050907	detection of chemical stimulus involved in sensory perception	3.27	9.17E-04
GO:0043112	receptor metabolic process	2.52	9.99E-04
**SMARCB1+ specific (gained) term:**			
GO:0072273	metanephric nephron morphogenesis	2.16	1.25E-13
GO:0003338	metanephros morphogenesis	2.03	1.90E-13
GO:0048643	positive regulation of skeletal muscle tissue development	2.17	3.14E-10
GO:0072207	metanephric epithelium development	2.09	3.17E-10
GO:0072170	metanephric tubule development	2.13	5.77E-10

Supplementary Fig. 1: A detailed characterization of differential open chromatin sites the MRT PDOs. (A) Box plot summarizing bulk log transformed transcripts per million (TPM) values of *SMARCB1* in different PDO models (n = 1). The median is used as the centre measurement for each box, which encloses the range between the first and third quartiles. Whiskers extend to the largest (or smallest) values no further than 1.5 times the inter-quartile range (IQR) from the box hinges. Source data are provided as a source data file; (B) Differential peaks in P103 were detected using a Wald test (FDR <0.05 and fold change ≥2)(n = 3); (C) Transcription factor motif analysis in control and SMARCB1+-specific OCRs (n = 2); (D) Top 5 most significant GO terms associated with the control and SMARCB1+ P103.

Supplementary Fig. 2

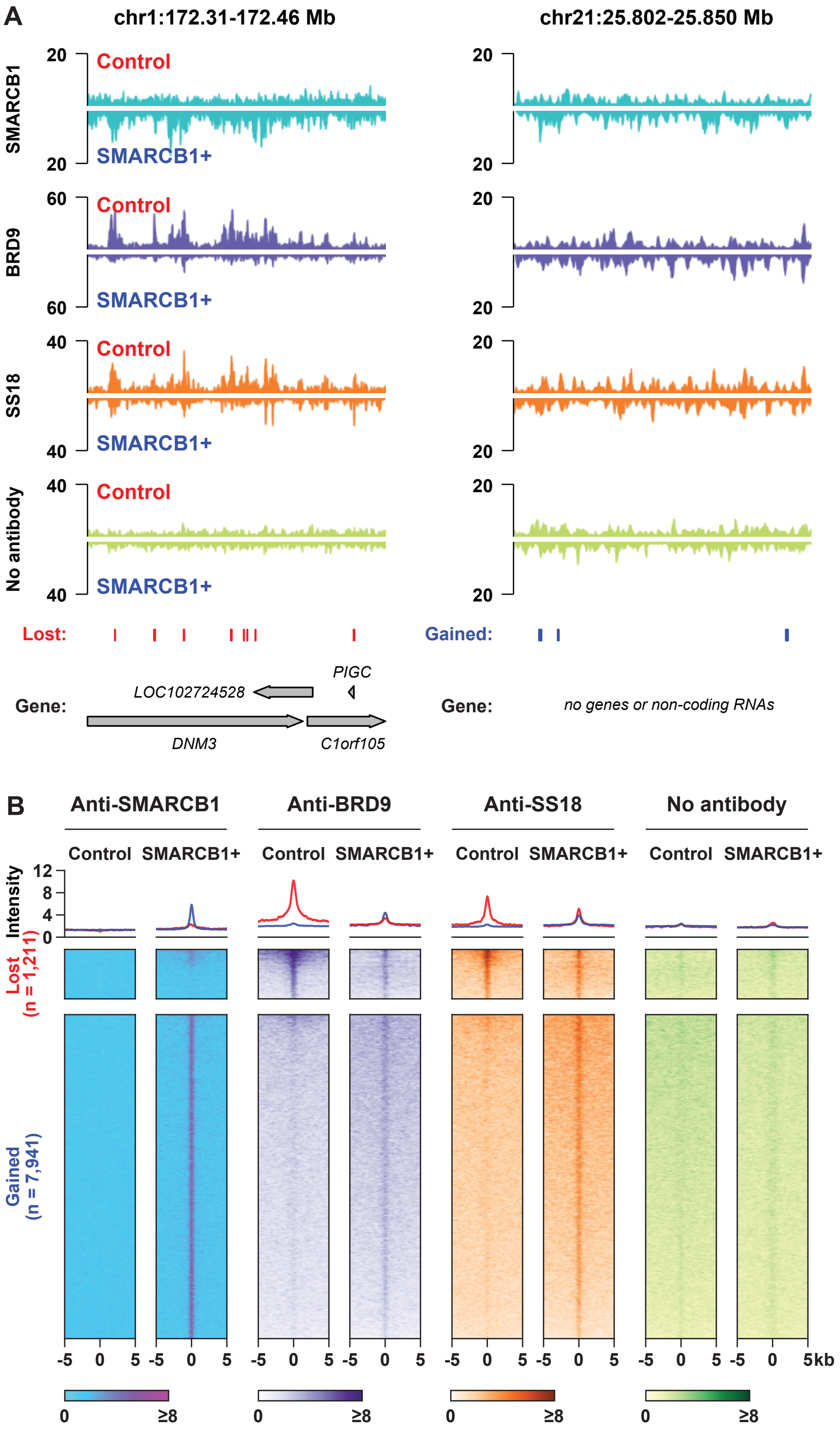

Supplementary Fig. 2: Characterization of BAF complex binding at differential open chromatin sites (A) Two example loci lost (left) and gained (right) chromatin accessibility after SMARCB1 reconstitution (as shown in Fig. 1B), respectively (CUT&RUN BRD9: n = 1,CUT&RUN SS18: n = 1; CUT&RUN no antibody control: n = 1); (B) Tornado-plot analysis showing that the lost (control-specific) open chromatin regions are losing BRD9 and SS18 binding after SMARCB1 reconstitution, while the gained (SMARCB1+-specific) open chromatin regions are increasing in SMARCB1 and SS18 binding (n = 1).

Supplementary Fig. 3

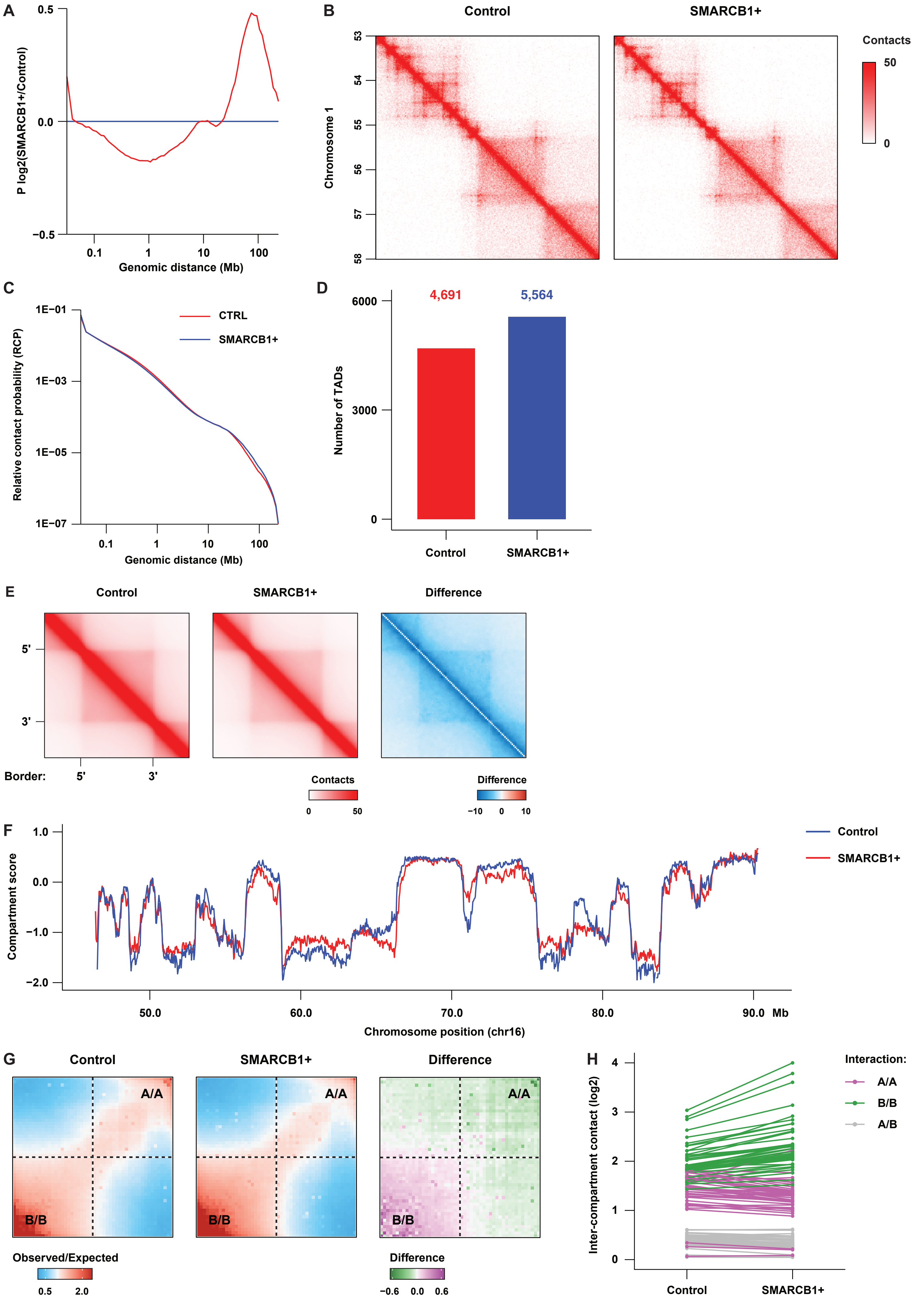

Supplementary Fig. 3: Impact of *SMARCB1* reconstitution on higher order of genome organization. (A) Relative contact probability in the Hi-C analyses before and after SMARCB1 reconstitution (n = 1); (B) An example locus exemplifying that TADs and loops are largely unaffected after SMARCB1 reconstitution; (C) Relative contact probably (RCP) of the Hi-C data from CTRL and SMARCB1+ P103 organoids. (D) A total number of TADs identified in the control and SMARCB1+ PDOs; (E) Aggregate TAD analysis (ATA) suggests that SMARCB1 reconstitution only has weak effects on chromatin contacts within TADs. Difference plot shows the subtraction of the Control versus SMARCB1 ATA plot; (F) Focal changes of A/B compartments after SMARCB1 reconstitution; (G,H) In contrast to the control cells, the SMARCB1+ cells show a higher frequency of B compartment interactions and a lower frequency of A compartment interactions.

Supplementarya Fig. 4

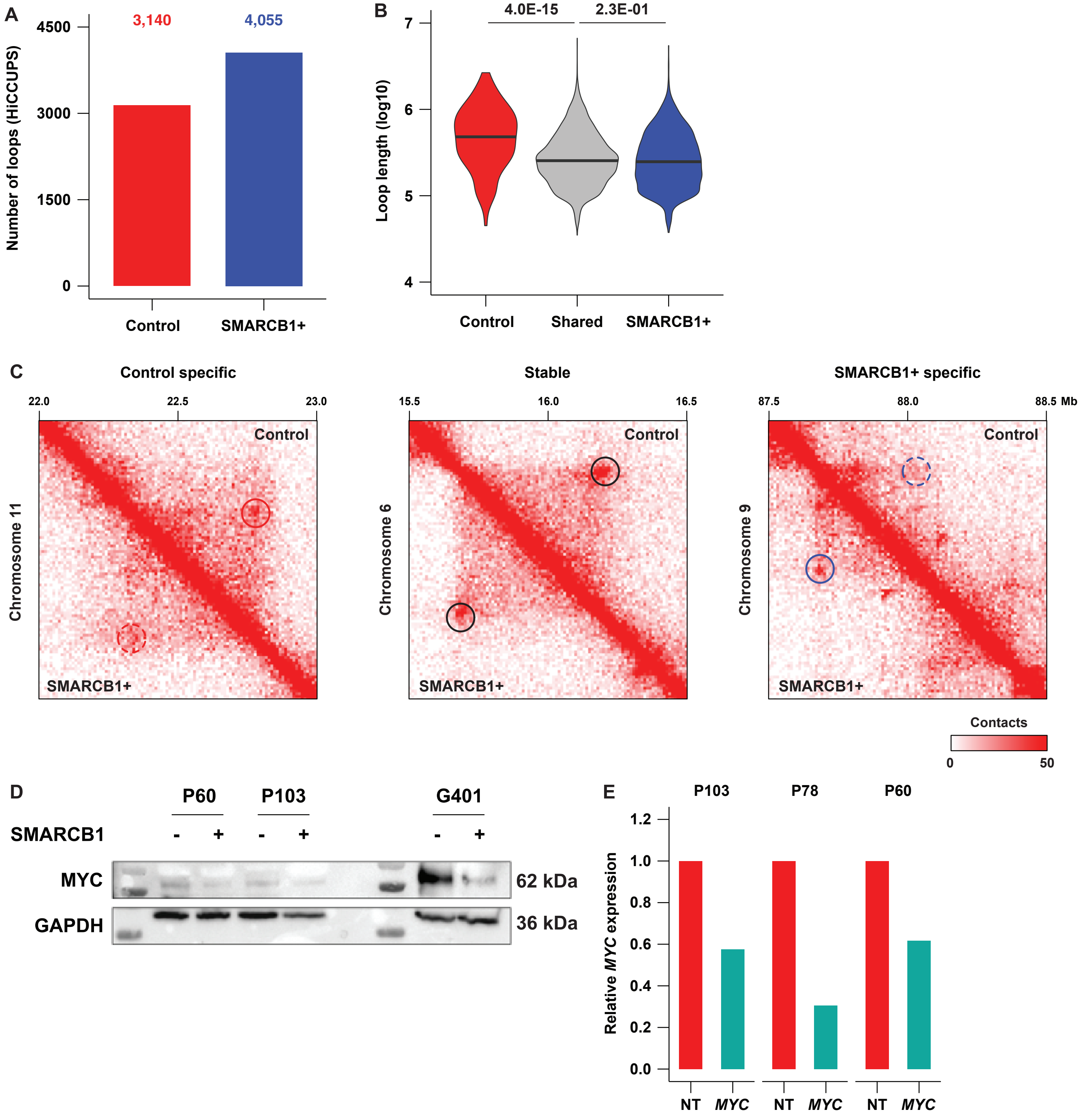

Supplementary Fig. 4: SMARCB1 dependent looping preference in MRT cells. (A) Total number of chromatin loops identified in the Control and *SMARCB1*+ cells of P103 PDO; (B) Violin plot depicting loop length comparing control-specific, SMARCB1+-specific and shared loops. Control-specific loops are significantly longer than other identified loops; (C) Three types of the identified loops based on their changes upon SMARCB1 reconstitution (Left: Control-specific loop; Middle: Stable loop; Right: SMARCB1+-specific loop).; (D) Western Blot of MYC protein expression after SMARCB1 reconstitution. GAPDH was used as loading control (n = 1)(MYC = 62kDa, GAPDH = 36kDa). Source data are provided as a source data file; (E) Relative *MYC* expression measured by RT-qPCR in the three organoid lines following shRNA *MYC* knockdown (n = 1). Source data are provided as a source data file.

Supplementary Fig. 5

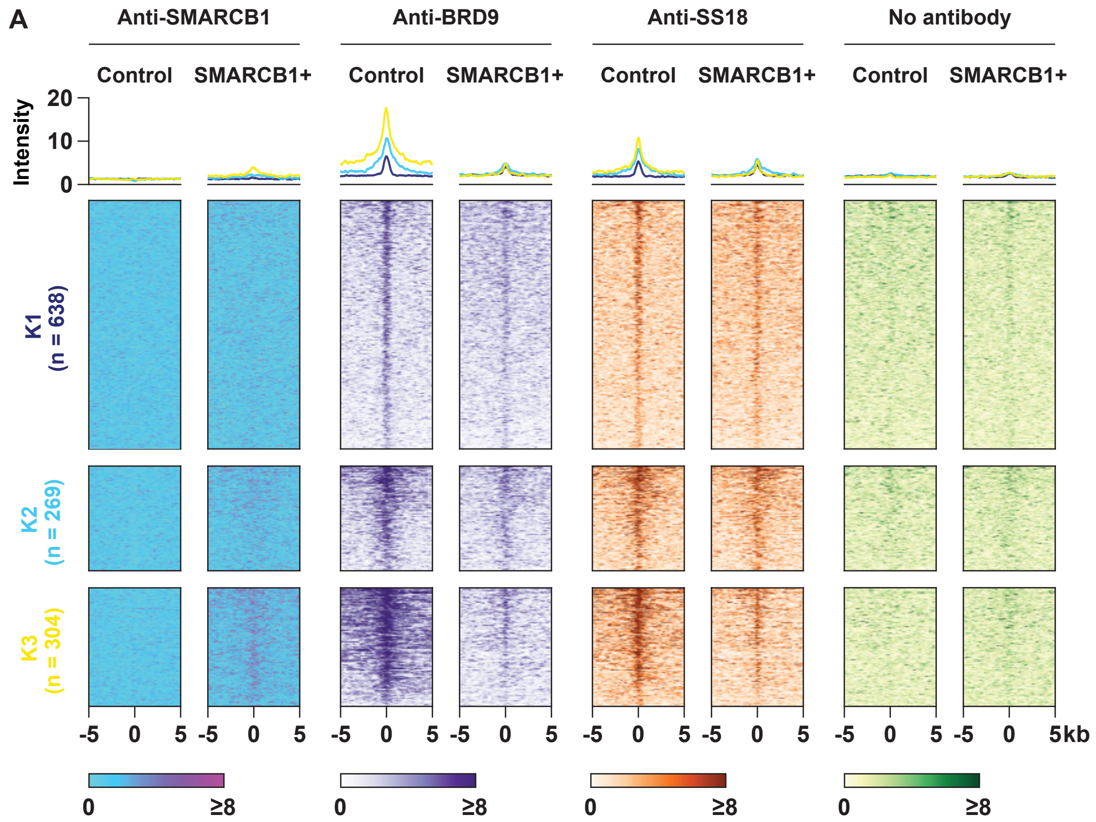

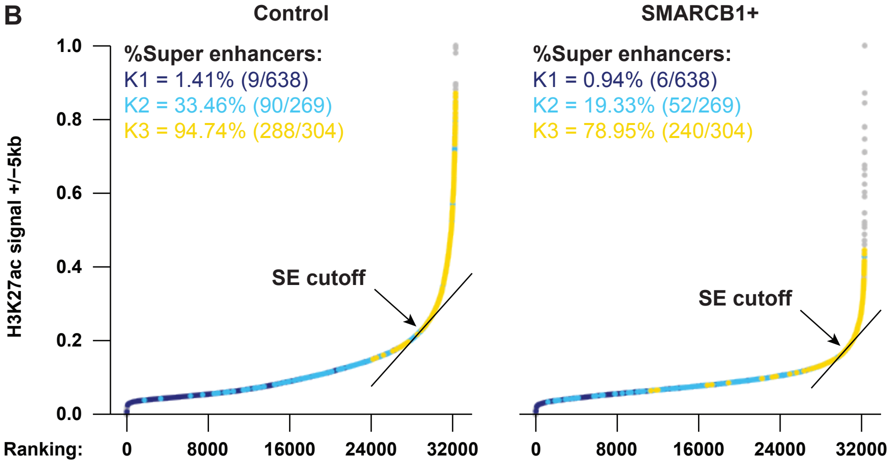

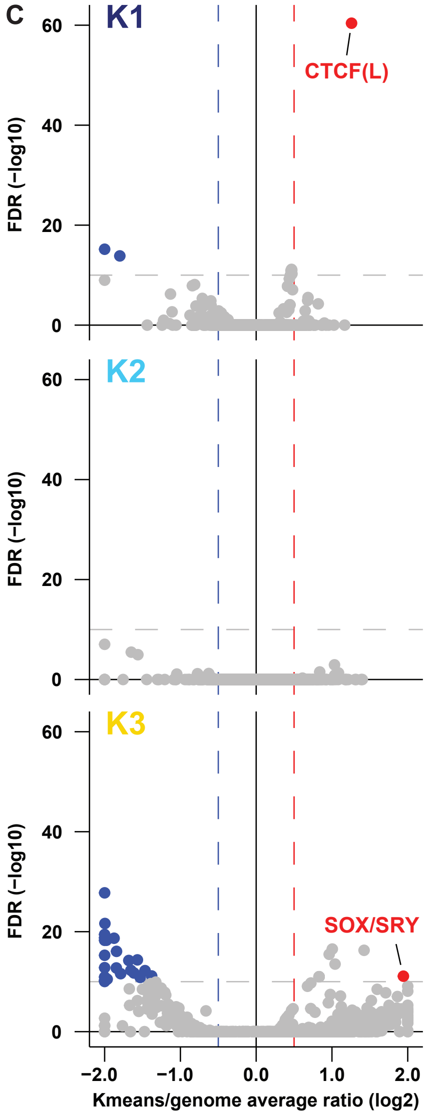

D Top 5 most significant biological processes (3 Kmeans clusters)

ID	Biological process	Fold change	FDR
**K1 specific GO term:**			
GO:0043271	negative regulation of ion transport	2.78	2.72E-02
**K2 specific GO term:**			
GO:0071824	protein-DNA complex subunit organization	4.76	6.16E-08
GO:0065004	protein-DNA complex assembly	5.04	2.97E-07
GO:0034728	nucleosome organization	5.36	2.51E-06
GO:0006334	nucleosome assembly	5.97	5.59E-06
GO:0031497	chromatin assembly	5.25	5.59E-06
**K3 specific GO term:**			
GO:0051056	regulation of small GTPase mediated signal transduction	3.88	2.82E-13
GO:0051241	negative regulation of multicellular organismal process	2.43	1.84E-12
GO:0051093	negative regulation of developmental process	2.43	7.51E-11
GO:0046578	regulation of Ras protein signal transduction	4.04	4.01E-10
GO:0022603	regulation of anatomical structure morphogenesis	2.29	8.06E-10

Supplementary Fig. 5: Molecular stratification of the common and patient-specific super enhancers in the MRT PDOs. (A) Additional tornado plots of K-means clustering analysis (Fig. 4A) showing increased SMARCB1 and reduced BRD9 and SS18 binding in the K3 cluster (n = 1); (B) Ranking H3K27ac signals of the three K-means clusters suggests that the majority of the K3 open chromatin sites are super enhancers; (C) Identification of transcription factor motifs that are overrepresented in the three types of open chromatin sites; (D) Top GO terms identified in each of the K-means clusters.

Supplementary Fig. 6

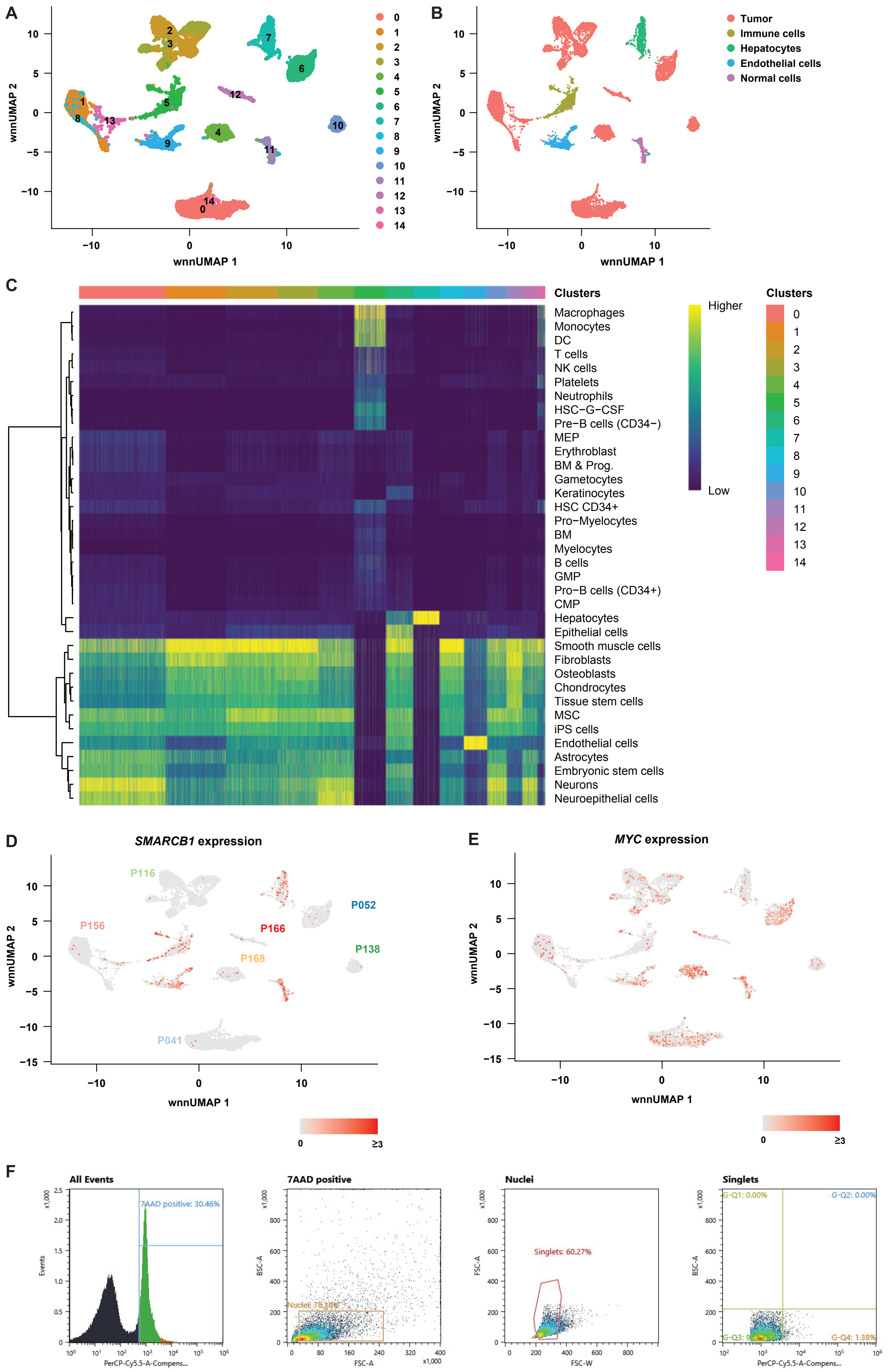

Supplementary Fig. 6: Identification of patient-specific molecular groups using single-cell multi-ome. (A) Single-cell multiome analysis identifies fifteen distinctive molecular clusters from seven MRT tumor tissues; (B,C) UMAP space and hierarchical clustering analysis discriminate different cell types within these tumor tissues based on RNA marker expression for certain cell types;(D,E) The expression levels of *SMARCB1* (D) and *MYC* (E) in the fifteen molecular clusters are visualized at single-cell level. (F) Example of the FACS gating strategy of 7ADD positive nuclei.

Supplementary Fig. 7

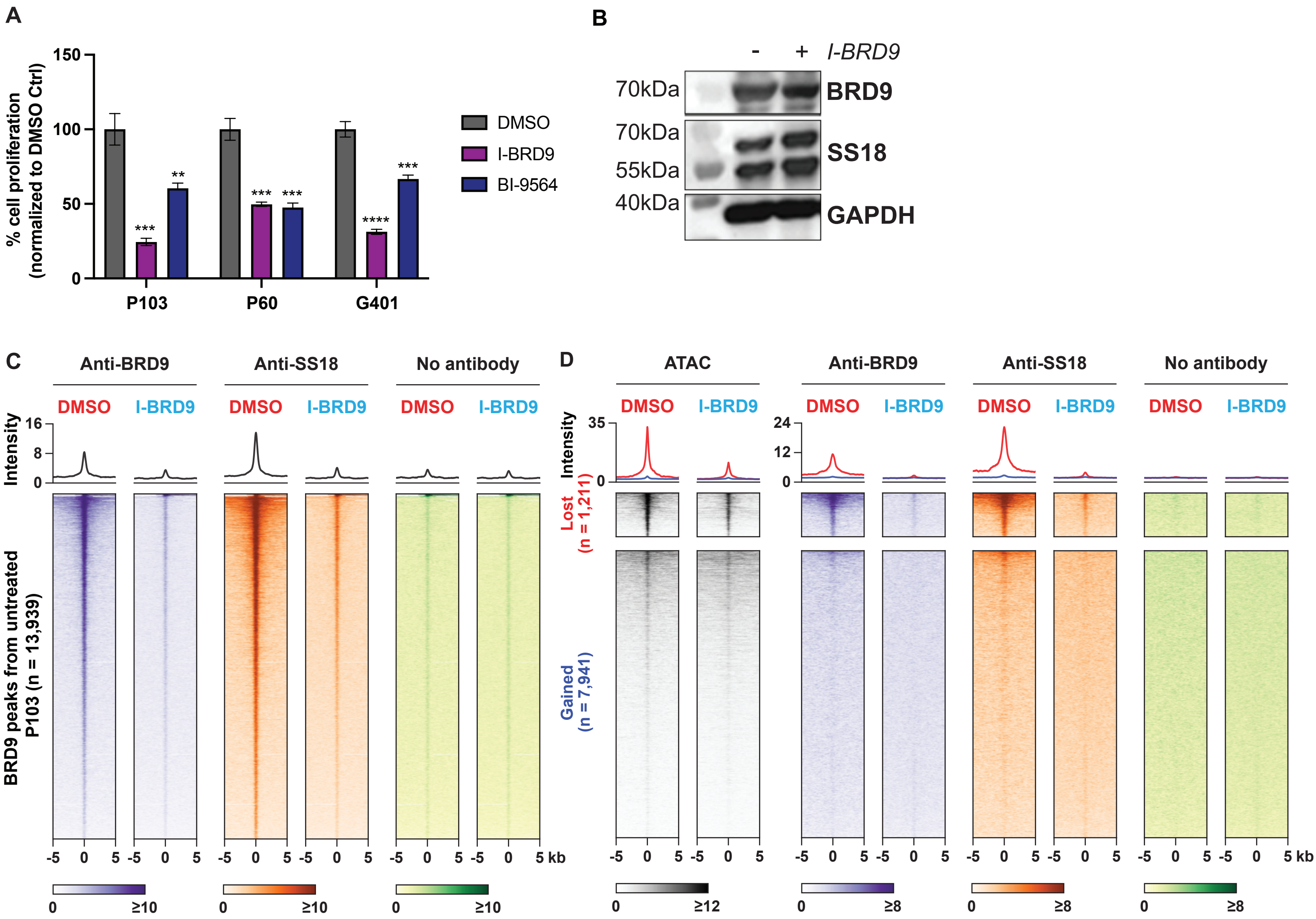

Supplementary Fig. 7: Chromatin occupancy of the SWI/SNF components before and after receiving I-BRD9 treatment. (A) CellTiter-Glo-assays to measure cell viability of the indicated MRT models treated with either vehicle (DMSO), I-BRD9 [10 µM], or BI-9564 [10 µM] for 120 hours (n = 3). Statistical significance was tested by unpaired two-sided t-test (*: p<= 0.05, **: p<= 0.01, ***: p<= 0.001, ****: p <= 0.0001). Source data are provided as a source data file; (B) Western Blot of BRD9 and SS18 after 5 days of I-BRD9 treatment [10 µM] in MRT PDOs (P103)(n = 1). GAPDH was used as a loading control. Size of protein ladder is depicted on the left side. Source data are provided as a source data file. (C) The binding of BRD9 and SS18 at BRD9 binding site with and without I-BRD9 treatment (n = 1); (D) Chromatin occupancy of active chromatin features and the SWI/SNF components at the lost and gained open chromatin sites identified in the SMARCB1 reconstitution experiments.

Supplementray Fig. 8

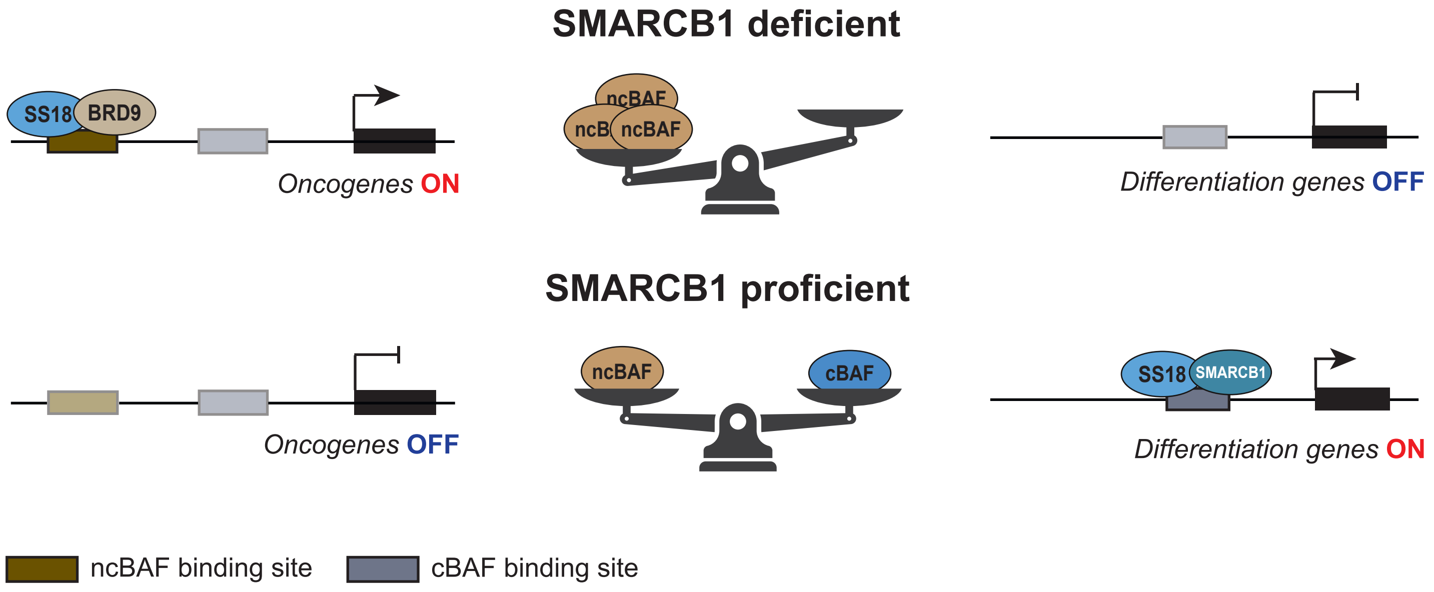

Supplementary Fig. 8: Schematic model of SMARCB1-dependent BAF complex re-distribution

Schematic model of the distribution of non-canonical (ncBAF) and canonical (cBAF) BAF complexes in SMARCB1 deficient(top) or proficient (bottom) cells. In the absence of SMARCB1, the BAF complex assembly is shifted towards the ncBAF composition leading to activated gene expression of, for instance, oncogenes. In SMARCB1 proficient cells, the cBAF complex competes with the ncBAF complex restoring the balance between the BAF complex compositions leading to re-distribution of the ncBAF complex and binding of the cBAF complex, thereby activating, for instance, expression of differentiation genes (Weighing scale icon adapted from Adobe Stock (@Max)).
