# Supplementary material for: SMARCB1 loss activates patient-specific distal oncogenic enhancers in malignant rhabdoid tumors
Source: Nat Commun. 2023 Dec 1;14:7762. doi: 10.1038/s41467-023-43498-3 (PMC10692191; doi:10.1038/s41467-023-43498-3)
Supplement: Supplementary file 3 — Description of Additional Supplementary Files [file 41467_2023_43498_MOESM3_ESM.pdf]

Description of Additional Supplementary Files

File Name: Supplementary Data 1
Description: Overview of PDO samples and sequencing techniques

File Name: Supplementary Data 2
Description: Ranked differential chromatin loops of HiC and their putative gene target

File Name: Supplementary Data 3
Description: Overview of used publicly available data sets.

File Name: Supplementary Data 4
Description: MRT tissues used for single-cell multiome analysis.
