# Supplementary material for: SMARCB1 loss activates patient-specific distal oncogenic enhancers in malignant rhabdoid tumors
Source: Nat Commun. 2023 Dec 1;14:7762. doi: 10.1038/s41467-023-43498-3 (PMC10692191; doi:10.1038/s41467-023-43498-3)
Supplement: Supplementary file 8 — Reporting Summary [file 41467_2023_43498_MOESM8_ESM.pdf]

nature portfolio

Corresponding author(s): Jarno Drost, Elzo de Wit

Last updated by author(s): Nov 6, 2023

Reporting Summary

Nature Portfolio wishes to improve the reproducibility of the work that we publish. This form provides structure for consistency and transparency in reporting. For further information on Nature Portfolio policies, see our Editorial Policies and the Editorial Policy Checklist.

Statistics

For all statistical analyses, confirm that the following items are present in the figure legend, table legend, main text, or Methods section.

n/a	Confirmed
☐	☒ The exact sample size (*n*) for each experimental group/condition, given as a discrete number and unit of measurement
☒	☐ A statement on whether measurements were taken from distinct samples or whether the same sample was measured repeatedly
☐	☒ The statistical test(s) used AND whether they are one- or two-sided<br>*Only common tests should be described solely by name; describe more complex techniques in the Methods section.*
☒	☐ A description of all covariates tested
☐	☒ A description of any assumptions or corrections, such as tests of normality and adjustment for multiple comparisons
☐	☒ A full description of the statistical parameters including central tendency (e.g. means) or other basic estimates (e.g. regression coefficient) AND variation (e.g. standard deviation) or associated estimates of uncertainty (e.g. confidence intervals)
☐	☒ For null hypothesis testing, the test statistic (e.g. *F*, *t*, *r*) with confidence intervals, effect sizes, degrees of freedom and *P* value noted<br>*Give P values as exact values whenever suitable.*
☒	☐ For Bayesian analysis, information on the choice of priors and Markov chain Monte Carlo settings
☒	☐ For hierarchical and complex designs, identification of the appropriate level for tests and full reporting of outcomes
☒	☐ Estimates of effect sizes (e.g. Cohen's *d*, Pearson's *r*), indicating how they were calculated

Our web collection on statistics for biologists contains articles on many of the points above.

Software and code

Policy information about availability of computer code

Data collection	ATAC-seq libraries were sequenced using the single-end 65-cycle mode on an Illumina HiSeq 2500 platform. ChIP-seq and RNA-seq libraries were sequenced using the single-end 65-cycle mode on an Illumina HiSeq 3000 or the paired-end 38×2 on a NextSeq 550. Hi-C libraries were prepared using a standard end-repair and A-tailing method and sequenced on an Illumina NovaSeq 6000 sequencer generating paired-end 150 bp reads. The 4C-seq libraries were sequenced using paired-end 76 cycles (44+32 cycles) on NextSeq 550 platform, and only the 44 bp end is used for analysis. CUT&RUN libraries were sequenced on an Illumina NextSeq2000 platform using paired-end 100 cycles (2x50bp).<br><br>MRT tissues were processed using standard tissue processing procedure following the 10X Genomics protocol. Between 2.000 to 40.000 nuclei were loaded on the Chip J Chromatin Controller (10X Genomics). Library preparation of Gene expression (GEX) as well as ATAC library was performed following manufacturer instructions (10X Genomics). Libraries were sequenced on the Illumina platform NovaSeq6000 paired-end according to manufacturer specifications (10X Genomics).
Data analysis	Hi-C sequencing data was processed with Hi-C-Pro (v 2.9.0). We performed loop calling with HICCUPs (v 0.9). Hi-C data analysis was performed with GENOVA (version: 1.0.0). ATAC-seq and CUT&RUN data were were mapped against hg38 reference genome using BWA-MEM (v 0.7.15-r1140), and analyzed using deeptools (v 3.0). Mapping of (double-crosslinking) ChIP-seq data was performed with bowtie2 (v 2.3.4.1) to hg38 genome. A mapping quality score of 15 was used to filter the mapped reads using samtools (version: 1.10). We performed peak calling with MACS2 (v 2.2.7.1) at a q-value cutoff of 0.01. ChIP-seq alignment plots were created with deeptools (v 3.5.1). RNA-seq data was mapped with TopHat (v 2.1.1) and count-tables were generated with HTseq (v 0.11.3) using the Homo Sapiens GRCm38.92 build. The read normalization was performed using DESeq2 (v 1.24.0). GSEA was performed using a desktop version of GSEA tool (v 4.1.0) and a Molecular Signatures Database (MSigDB, v2022.1). Motif analysis was done by GimmeMotifs (v 0.16.1) using the non-redundant cis-bp database (v 3.0). A web-version GREAT analysis tool was used to annotate the ATAC-seq peaks (version 4.0.4). The raw 4C-seq data was mapped using our 4C mapping pipeline (http://github.com/deWitLab/4C_mapping). We normalized our 4C data to 1 million intrachromosomal reads using peakC (version:
